# Fire Extinguishing Performance of Chemically Bonded Struvite Ceramic Powder with High Heat-Absorbing and Flame Retardant Properties

**DOI:** 10.3390/ma15228021

**Published:** 2022-11-14

**Authors:** Zilong Liang, Zhiji Zhou, Yunqi Sun, Yujia Huang, Xinya Guo, Guoshuai Cai, Mingchao Wang, Haijun Zhang

**Affiliations:** 1 School of Safety Science and Engineering, Civil Aviation University of China, Tianjin 300300, China; 2 College of Science, Civil Aviation University of China, Tianjin 300300, China

**Keywords:** chemically bonded ceramic, struvite, recycling, ultrafine powder, fire extinguishing performance

## Abstract

Struvite is a chemically bonded ceramic product in the pipeline of a sewage treatment plant. In order to explore the fire extinguishing potential of struvite, a new type of struvite ultrafine dry powder with excellent performance was prepared by a simple process, and its fire extinguishing performance and mechanism were analyzed in depth. Under the same process conditions, the refinement degree (D50 = 5.132 μm) and the specific surface area (BET = 25.72 m^2^/g) of ultrafine struvite were larger than those of NH_4_H_2_PO_4_ (D50 = 8.961 μm, BET = 13.64 m^2^/g), making struvite more suitable for fire extinguishing. Besides, the pyrolysis process of struvite was relatively concentrated and absorbed more heat in a short time. Its heat absorption (458.4 J/mg) was higher than that of NH_4_H_2_PO_4_ (156.4 J/mg). Water, ammonia, and PO· were released during the pyrolysis of struvite, which effectively reduced fire temperature, diluted oxygen concentrations and captured free radicals. At the same time, the final products were magnesium orthophosphate and magnesium pyrophosphate, which formed a dense flame-retardant ceramic layer with good thermal insulation and environmental protection functions. In these cases, the fire extinguishing mechanism of struvite was determined to have three stages: the cooling effect, the asphyxiation effect, and the chemical effect. Correspondingly, the fire extinguishing time of struvite was three seconds faster than that of ammonium phosphate under 0.2 MPa based on the local oil basin test.

## 1. Introduction

The particle size of extinguishing components used in ordinary dry powder is usually between 10~75 μm. Due to the relatively large mass of a single particle, it has the disadvantages of fast sedimentation, poor dispersion, and a small specific surface area, which in turn leads to a relatively poor ability to capture free radicals and active groups and limits fire extinguishing ability [1]. The preparation of ultrafine dry powder with smaller particle size, good fluidity, a large specific surface area, high activity, stable dispersion, and the ability to be suspended in the air for a long time is the key to improving the fire extinguishing ability of dry powder [2]. In recent years, ultrafine dry powder has attracted the attention of scholars and fire-fighting enterprises because of its excellent fire extinguishing performance [3]. The development of new materials has become one of the research hotspots for ultrafine dry powder [4].

Magnesium ammonium phosphate, also known as struvite (MgNH_4_PO_4_ · 6H_2_O) is a naturally occurring orthophosphate mineral [5]. Due to the high concentration of ammonium and phosphate in the water system of the sewage treatment plant, struvite crystals are easily formed in the pipeline [6]. If the pipeline is not cleaned for a long time, the crystal will block the pipeline, so a large amount of struvite will be recycled every year. In order to reduce the cost of waste treatment and improve the efficiency of chemical treatment processes, alternative uses of this material need to be found [7]. Struvite is rich in nitrogen and phosphorus, and its solubility is low (169.2 mg/cm^−3^ at 25 °C), which can be used as a slow-release fertilizer [8]. However, in the process of recycling struvite in sewage treatment plants, some heavy metals and toxic substances are incorporated, which limits its application in agriculture [9,10]. It is found that struvite is a chemically bonded phosphate-containing ceramic [11,12,13], which can be transformed from an unstable chemically bonded ceramic to a stable heat-resistant ceramic during heating by releasing large amounts of gas and active groups [14,15,16,17,18,19]. Researchers in the field of building materials have mixed struvite into cement to improve its corrosion and high temperature resistance [20]. By mineralizing struvite on the surface of wood, Guo et al. [21,22] found that struvite absorbs heat at high temperatures and releases phosphorus-containing substances that can form a carbon layer with carbon-containing materials. At the same time, thermal engine products will form a dense heat-insulating ceramic layer on the surface, which makes the wood have good flame-retardant properties. Struvite has gradually attracted the attention of scholars due to its excellent flame retardant and endothermic properties. There is great potential in the field of fire fighting. Therefore, it is of great significance to explore its application in fire extinguishing.

Struvite and ABC dry powder (NH_4_H_2_PO_4_) have similar pyrolysis mechanisms. When the struvite is decomposed by heating, a large amount of water vapor, ammonia, and phosphorus oxygen groups are released [23], which can not only reduce the actual temperature of the fire field but also dilutes the concentration of oxygen and combustible gas near the flame. Moreover, phosphorus oxygen groups can also consume the free radicals in the combustion reaction, so that the number of free radicals decreases sharply, resulting in the interruption of the combustion reaction chain. At the same time, the decomposition product (Mg_2_P_2_O_7_) is a good refractory ceramic material, which covers the surface of the combustible, thus playing a role in isolating air and preventing reburning. Its potential in the field of fire protection is supposed to be further explored. Although the pyrolysis mechanisms of struvite and NH_4_H_2_PO_4_ are similar, the pyrolysis products of struvite are stable neutral oxides, which are not corrosive to equipment and are more environment-friendly. Therefore, struvite has a great advantage as a fire extinguishing agent. In this work, ultrafine struvite powder was synthesized using an economic method. By comparing the particle size distribution, microstructure, pyrolysis, and fire extinguishing experimental data of ultrafine struvite powder and ultrafine NH_4_H_2_PO_4_ powder, the fire extinguishing mechanism was explained in detail.

## 2. Experimental Methods

### 2.1. Materials

All reagents (AR) were purchased from chemical reagent companies. Nano-hydrophobic silica and struvite were purchased from Shanghai McLin Biochemical Technology Co., Ltd. (Shanghai, China). Ammonium dihydrogen phosphate and anhydrous ethanol were purchased from Shanghai Aladdin Biochemical Technology Co., Ltd. (Shanghai, China).

### 2.2. Sample Preparation

**Figure 1:**
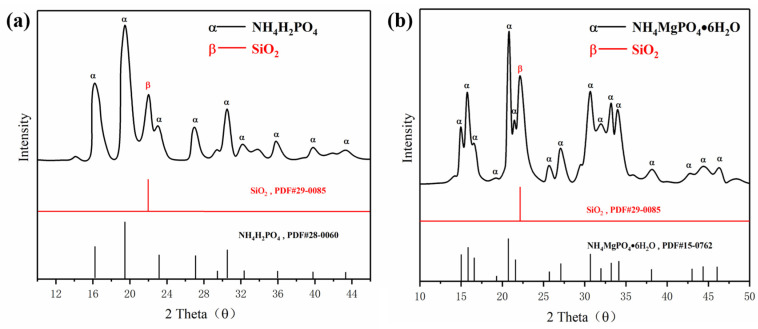
XRD analysis of (**a**) NH_4_H_2_PO_4_ powder and (**b**) struvite.

The preparations of ultrafine struvite powder and ultrafine NH_4_H_2_PO_4_ powder were prepared as follows: firstly, the struvite was crushed and refined by a horizontal planetary ball mill; the time and speed were controlled at 40 min and 400 r/min. The ratio of grinding ball, struvite, and absolute ethanol was controlled at 5:1:1. The refined struvite was then dried at room temperature for 5 h. Finally, 5 wt.% of hydrophobic silica was added to the dried sample for secondary ball milling to obtain an ultrafine struvite powder. The preparation of ultrafine NH_4_H_2_PO_4_ powder was the same as the above preparation conditions. Figure 1 shows the XRD spectra of the two synthesized materials. As shown in Figure 1a,b, the crystal peak of silica is identified at 2 theta of 22.03°, and no other new substances are identified on the two spectra, which confirmed that hydrophobic silica is only mixed with two samples and does not react to form new substances. In these cases, the mixed hydrophobic silica gives both samples good fluidity and hydrophobicity to meet fire extinguishing agent requirements.

### 2.3. Characteristic Method

Particle size distribution of samples measured with a laser particle size analyzer (LS-909, OMEC, Zhuhai, China). The BET analysis was performed using a specific surface and porosity analyzer (ASAP 2460), and nitrogen adsorption and desorption were performed at 100 °C for 8 h. Imaging analysis of samples was performed usng a scanning electron microscope (SEM, Antimony Lead Alloy 8100, Hitachi, Tokyo, Japan) with magnifications of 5.00 K and 50.0 K. Analysis of material composition used X-ray diffraction (XRD, Ultima IV, Rigaku, Tokyo, Japan); its parameters are set as follows: conventional wide-angle measurement (10–60°), test rate (10°/min). TG and DSC curves were obtained by a simultaneous thermal analyzer (STA449F3, Germany), and the pyrolysis process was analyzed. Its parameters are set as follows: test temperature (30–800 °C), heating rate (10 °C/min), gas environment (N_2_). Additionally, the characteristics of the two ultrafine dry powders, such as D50, D90, bulk density, fluidity, contact angle, consumption, fire extinguished time, were tested using a Rooko FT-2000A powder characteristic analyzer.

### 2.4. Fire Extinguishing Experiment

**Figure 2:**
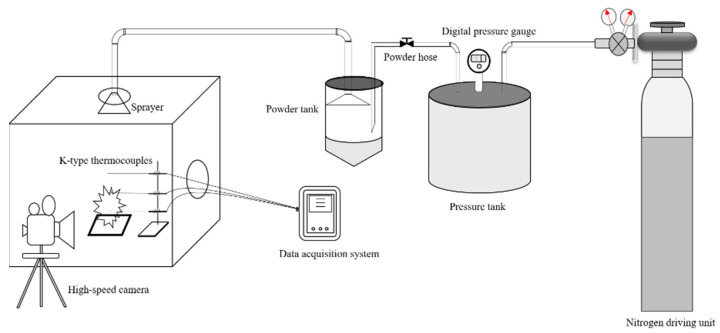
The experimental apparatus for the fire-extinguishing tests.

In order to compare the fire extinguishing performance of ultrafine NH_4_H_2_PO_4_ powder and ultrafine struvite powder, a small powder fire extinguishing device was used to carry out local oil basin tests [24,25,26,27]. The small powder fire extinguishing device used in the experiment is composed of three parts: a powder storage pressure device that is fire extinguishing, a temperature measuring device, and a data acquisition system. The schematic diagram of the experimental device is shown in Figure 2. The fuel fire used in the fire extinguishing experiment is a liquid pool fire produced by combining 200 mL of water with 150 mL of n-heptane in a 400 mm × 400 mm × 50 mm oil pan. The oil pan can be placed on iron cube supports of different heights to adjust the distance between the oil surface and the nozzle. The temperature acquisition system consists of three K-type thermocouples with a diameter of 4 mm connected to the data acquisition instrument to collect the flame temperature. The powder pressurizing device consists of a nitrogen bottle, a pressure tank, a powder tank, and a stainless-steel conical nozzle fixed to a stand with adjustable height and direction. The fire extinguishing time of different powders is recorded by an electronic stopwatch. During the fire extinguishing experiment, 50 g fire extinguishing agent were placed in powder cans as a fixed amount for the experiment, and the powder consumption was obtained by weighing the mass difference of the sample before and after the experiment with an electronic balance. In order to stabilize the flame temperature zone, each experiment required pre-burning for 60 s before releasing the sample. Each set of samples was repeated three times to reduce accidental errors.

## 3. Results and Discussion

### 3.1. Micromorphology Characterization

**Figure 3:**
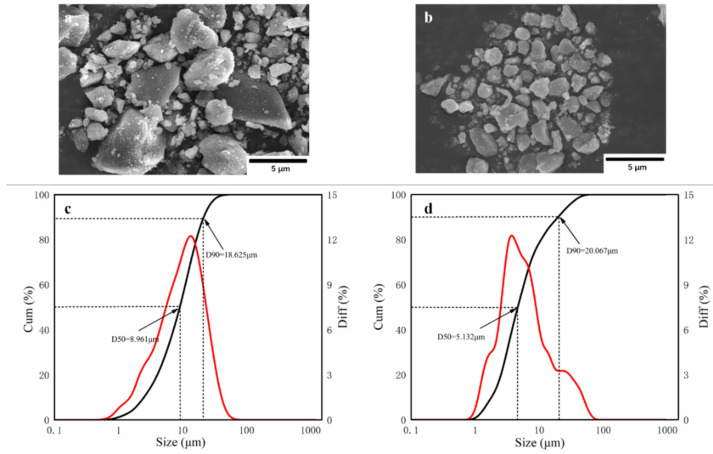
Scanning electron microscopy (SEM) images of (**a**) ultrafine NH_4_H_2_PO_4_ powder and (**b**) ultrafine struvite powder; Particle size and size distribution of (**c**) ultrafine NH_4_H_2_PO_4_ powder and (**d**) ultrafine struvite powder.

The morphology and particle size of the powder are the key factors affecting its fire-extinguishing effect. It is thought that when 90% of the particles in the powder are less than 20 μm (D90 < 20 μm), the powder achieves ultrafine requirements. As shown in Figure 3a,b, struvite and NH_4_H_2_PO_4_ meet the ultrafine requirements under the same process conditions, but they show different microstructures. Figure 3a shows that the ultrafine NH_4_H_2_PO_4_ powder exists in the form of an irregular prismatic structure, and there is an obvious agglomeration phenomenon in which the large particles are surrounded by small particles. Figure 3b shows that the ultrafine struvite powder exists in a relatively smooth columnar structure. The overall particle size is smaller, the particle size distribution is relatively uniform, and the agglomeration phenomenon is obviously improved. According to Figure 3c,d, the D90 of ultrafine NH_4_H_2_PO_4_ powder and ultrafine struvite powder are 18.625 μm and 20.077 μm, respectively, which reach the ultrafine standard. By comparing the average particle size (D50) of the two samples, it is found that the average particle size of struvite is smaller (D50 = 5.132 μm < 8.961 μm). The results show that struvite particles prepared under the same process conditions have smaller particle sizes and less agglomeration, indicating that struvite is not easily agglomerated in storage and has better dispersion and a faster decomposition rate in the fire, resulting in better fire extinguishing capacity.

**Figure 4:**
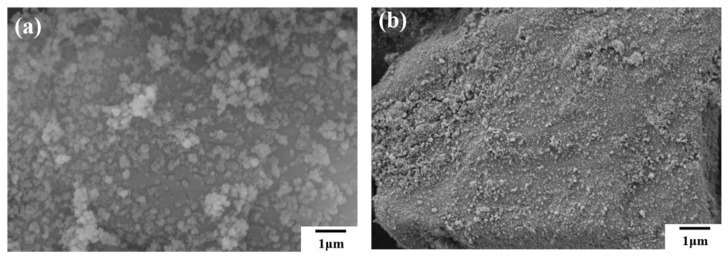
High magnification SEM images of (**a**) NH_4_H_2_PO_4_ powder and (**b**) struvite.

From the high-magnification SEM images of the two samples in Figure 4, it can be seen that nano-scale hydrophobic silica adheres to the surface of large particles, which solves the large particle agglomeration problem. At the same time, the silica-coated samples meet the fluidity and hydrophobicity requirements of fire extinguishing agents.

**Figure 5:**
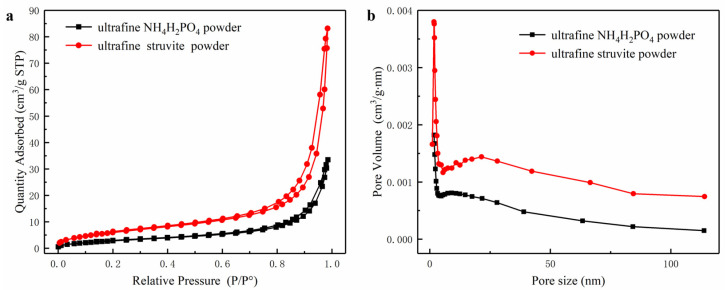
(**a**) Pore size distribution of samples, (**b**) Adsorption and desorption curves of samples.

Figure 5a,b show the adsorption-desorption curves and pore size distribution curves of the two powders. The specific surface area (25.72 m^2^/g) of ultrafine struvite powder is larger than that of ultrafine NH_4_H_2_PO_4_ powder (13.64 m^2^/g) (see Table 1). This phenomenon is most likely caused by the particle size and particle size distribution of the two samples. As the particle size becomes smaller, the specific surface area becomes larger. According to previous studies [3,4], particles with a large specific surface area capture free radicals in the fire field more easily, and the degree of pyrolysis is more thorough due to the increased contact area with the flame. Therefore, struvite is more suitable for fire extinguishing due to its uniform size distribution and relatively high surface area.

### 3.2. Fire Extinguishing Performance

**Figure 6:**
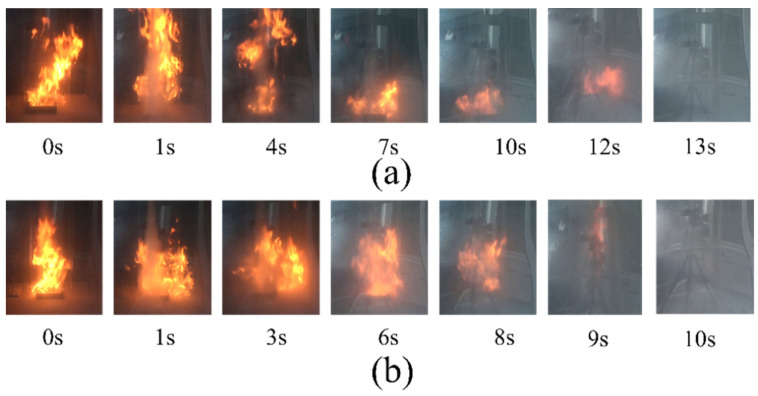
Images of the fire suppression processes of the tests with different samples at 0.2 MPa. (**a**) ultrafine NH_4_H_2_PO_4_ powder and (**b**) ultrafine struvite powder.

The fire extinguishing time of the two samples is less than 30 s under a 0.2 MPa nitrogen drive. However, the flame cannot be extinguished within 30 s with only 0.2 MPa nitrogen, which proves that nitrogen is not the main factor affecting flame extinction [4]. The fire-extinguishing performance of the two samples is different under the same driving pressure. The data from Table 2 show that struvite only takes 10 s to extinguish the flame at 0.2 MPa nitrogen pressure, while NH_4_H_2_PO_4_ takes more time (13 s). Besides, the consumption of struvite (13.067 g) is less than that of NH_4_H_2_PO_4_ (18.625 g). This result strongly shows that the extinguishing performance of struvite is better than that of NH_4_H_2_PO_4_. Figure 6 shows a screenshot of a fire extinguishing video of two samples under 0.2 MPa (the specific video of local oil basin experiments is shown in the Supplementary Materials).

**Figure 7:**
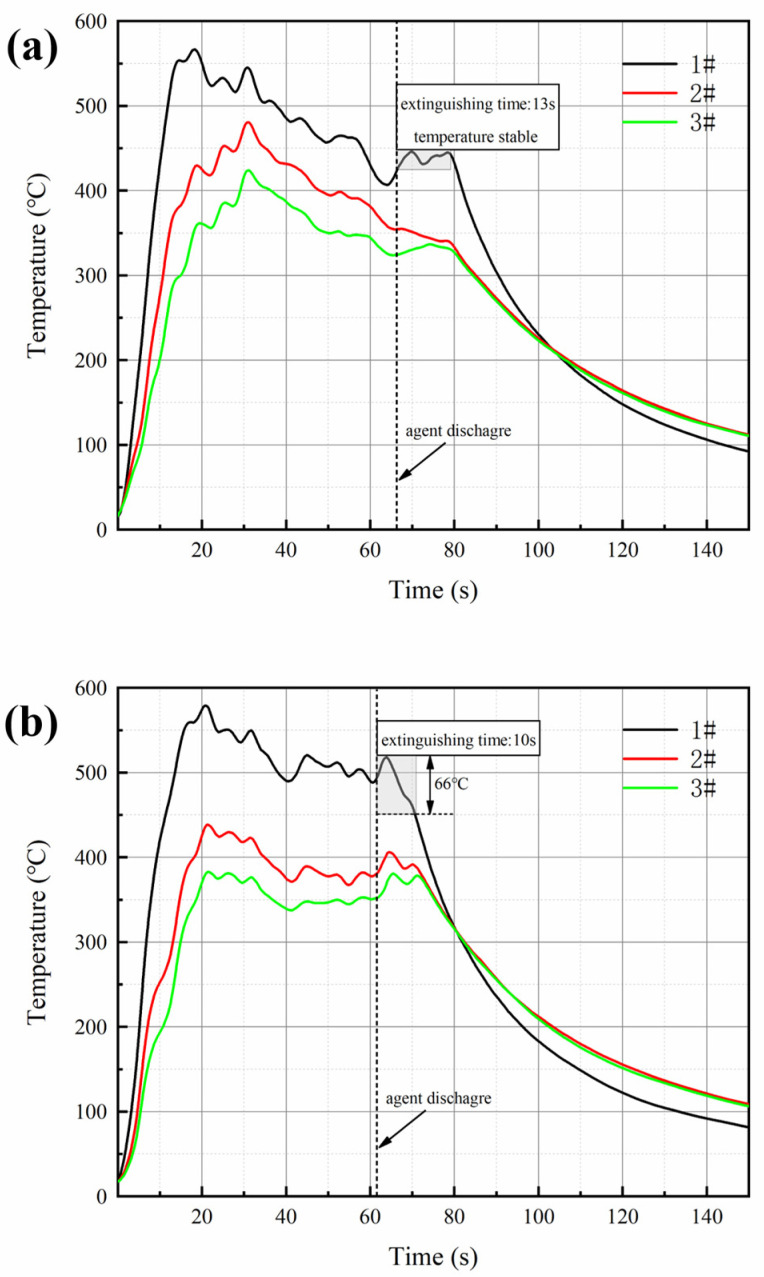
Temperature variation of (**a**) ultrafine NH_4_H_2_PO_4_ powder and (**b**) ultrafine struvite powder.

**Figure 8:**
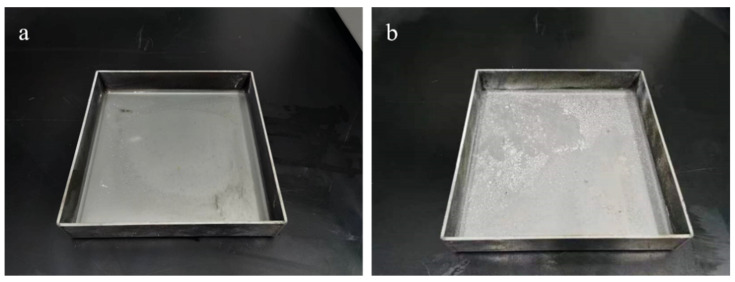
Fire extinguishing agent coverage effect: (**a**) ultrafine NH_4_H_2_PO_4_ powder and (**b**) ultrafine struvite powder.

**Figure 9:**
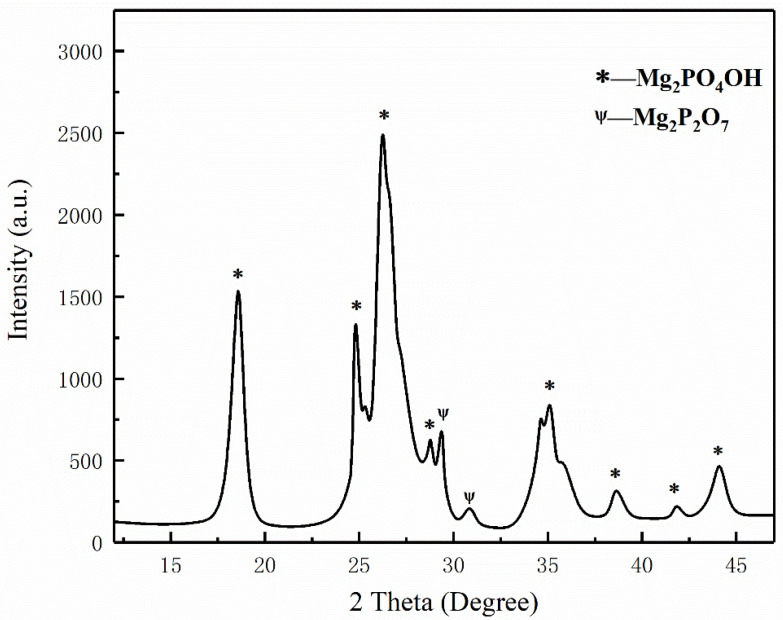
XRD analysis of pyrolysis products of struvite.

**Figure 10:**
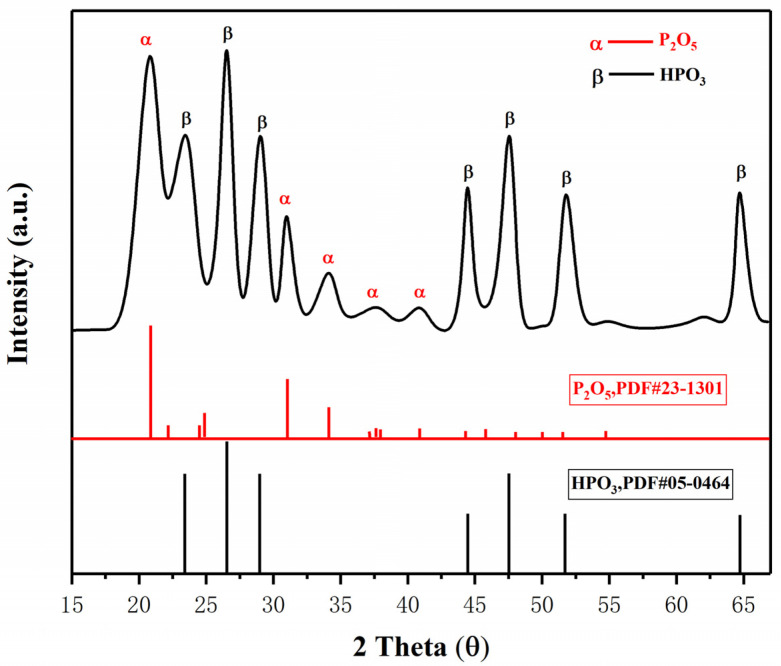
XRD analysis of pyrolysis products of NH_4_H_2_PO_4_.

Figure 7a,b show the temperature change of ultrafine NH_4_H_2_PO_4_ powder and ultrafine struvite powder after entering the fire (0.2 MPa). In order to stabilize the flame temperature zone, each experiment required pre-burning for 60 s before releasing the sample. When the flame was stabilized at 450–550 °C, the extinguishing agent was released into the fire. Due to the different fire-extinguishing characteristics of the two samples, the temperature change curves also show different trends. As shown in Figure 7a, the flame temperature rises briefly after the ultrafine NH_4_H_2_PO_4_ powder enters the fire field, and then the temperature fluctuates slightly. The temperature remains almost stable throughout the fire extinguishing period. As the flame is extinguished, the temperature of the fire suddenly drops. From Figure 7b, it can be seen that after the ultrafine struvite powder was released to the fire site, the temperature also increased briefl, and then decreased linearly. During the entire fire extinguishing time, the temperature drops by 66 °C. After the flame is extinguished, the temperature drops faster. By analyzing these two different temperature trends, it is strongly confirmed that struvite absorbs heat during pyrolysis in the fire field, thus reducing the flame temperature to achieve the purpose of rapidly extinguishing the fire. Figure 8 shows the covering effect of two samples after fire extinguishing. Compared with NH_4_H_2_PO_4_, the pyrolysis products of struvite form a larger area of coverage on the surface of the oil basin, effectively preventing the fuel from contacting oxygen to achieve a suffocating effect. At the same time, the covering layer is a stable heat-resistant ceramic (Mg_2_P_2_O_7_, Mg_2_PO_4_OH) shown in Figure 9, which will have a certain heat insulation effect. As shown in Figure 10, the remaining materials after NH_4_H_2_PO_4_ decomposition are P_2_O_5_ and incompletely decomposed HPO_3_, which easily combine with water molecules to form acidic substances that corrode the equipment. Under the synergy of many aspects, struvite shows a more efficient fire-extinguishing effect.

### 3.3. Comprehensive Characteristics of Powder

The comprehensive characteristics of two ultrafine powders are shown in Table 3. Where, D50 (μm) is the corresponding particle size when the cumulative particle size distribution percentage of the sample reaches 50%. D90 (μm) is the corresponding particle size when the cumulative particle size distribution percentage of the sample reaches 90%. Bulk density (g/cm^3^) refers to the ratio of the mass of the powder to its filling volume (including the gap between the dry powder) when the sample is not vibrated. Fluidity (g/s) refers to the time required for a sample to flow through a standard funnel with a prescribed aperture in a certain amount of dry powder. Contact angle (°) refers to the angle (θ) between the sample solid-liquid interface and is a measure of the degree of hydrophobicity.

By comparison, the ultrafine struvite powder prepared in this paper meets the ultrafine requirements (D90 < 20 μm), and all the bulk density, fluidity, and contact angle reach the level of commercial dry powder. Moreover, its fire extinguishing time and fire extinguishing dosage are obviously better than ultrafine NH_4_H_2_PO_4_ powder.

### 3.4. Fire-Extinguishing Mechanism Discussion

**Figure 11:**
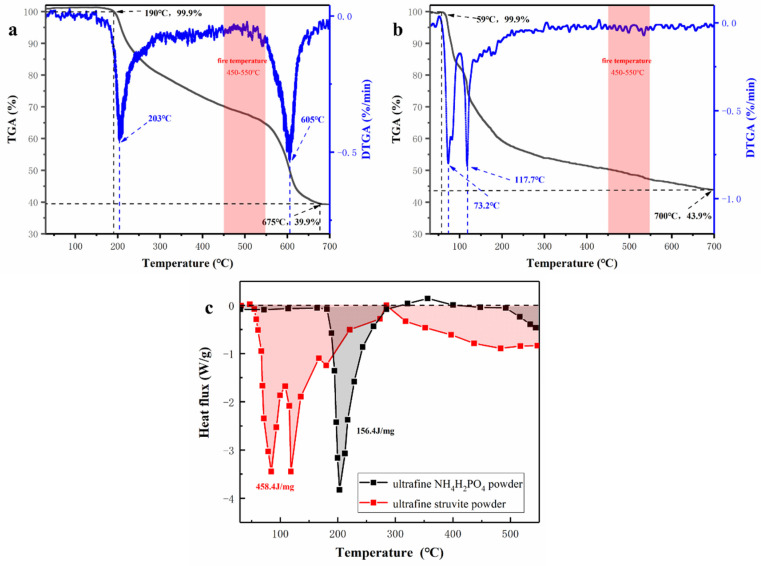
(**a**) Thermogravimetric (TG) and Derivative thermogravimetric (DTG) curves of NH_4_H_2_PO_4_, (**b**) Thermogravimetric (TG) and Derivative thermogravimetric (DTG) curves of struvite, (**c**) Differential scanning calorimetry (DSC) curves of sample powders.

In the process of fire extinguishing, the thermal decomposition characteristics of dry powder have a very important influence on its fire extinguishing efficiency, so the decomposition process of samples in the experiment was analyzed. TG-DSC curve, combined with the appearance characterization and fire extinguishing performance of the two samples, explain the fire extinguishing mechanism of struvite. According to the curve of Figure 11, it is found that struvite begins to pyrolyze at 60 °C; the pyrolysis process is relatively concentrated (60–150 °C), which mainly releases large amounts of crystal water and ammonia and absorbs lots of heat in a short time. Then the struvite is slowly pyrolyzed at 200–700 °C and gradually transformed into a stable heat-resistant ceramic (Mg_2_P_2_O_7_), which is a stable neutral oxide and forms a dense thermal insulation layer covering the surface of the oil to isolate the air. The gas-phase phosphorus-oxygen groups generated during this period can capture the free radicals. Ultrafine NH_4_H_2_PO_4_ powder has two decomposition processes: the first stage begins at 190 °C (releasing water and ammonia gas), and the second stage begins at 550 °C and produces P-O groups and unstable P_2_O_5_, which easily converts phosphoric acid (corrosion to equipment) in water. Because the flame temperature is stable at 500 °C during the experiment, the secondary pyrolysis of NH_4_H_2_PO_4_ cannot be realized, resulting in limited fire extinguishing ability. Ultrafine struvite powders contact the flame more fully because of their smaller particle size and larger specific surface area. As seen from the DTG curve, struvite has a faster decomposition rate due to its smaller particle size. Although the decomposition residue of struvite (43.9%) is higher than that of NH_4_H_2_PO_4_ (39.9%), this is related to the characteristics of the material itself. The heat absorption of the ultrafine struvite powder is 458.4 J/mg, which is significantly larger than that of the ultrafine NH_4_H_2_PO_4_ powder (156.4 J/mg) from Figure 11c. It can be inferred that struvite absorbs more heat to reduce the fire temperature after entering the fire site. The temperature change curve in Figure 7 can well demonstrate this conclusion. Therefore, struvite has better fire extinguishing efficiency in terms of pyrolysis characteristics.

The pyrolysis process of NH_4_H_2_PO_4_ is as follows [28]:

NH_4_H_2_PO_4_ → NH_3_ + H_3_PO_4_ (160 °C) (1)

H_3_PO_4_ → H_4_P_2_O_7_ + H_2_O (220 °C) (2)

H_4_P_2_O_7_ → HPO_3_ + H_2_O (360 °C) (3)

HPO_3_ → P_2_O_5_ +H_2_O (500 °C) (4)

**Figure 12:**
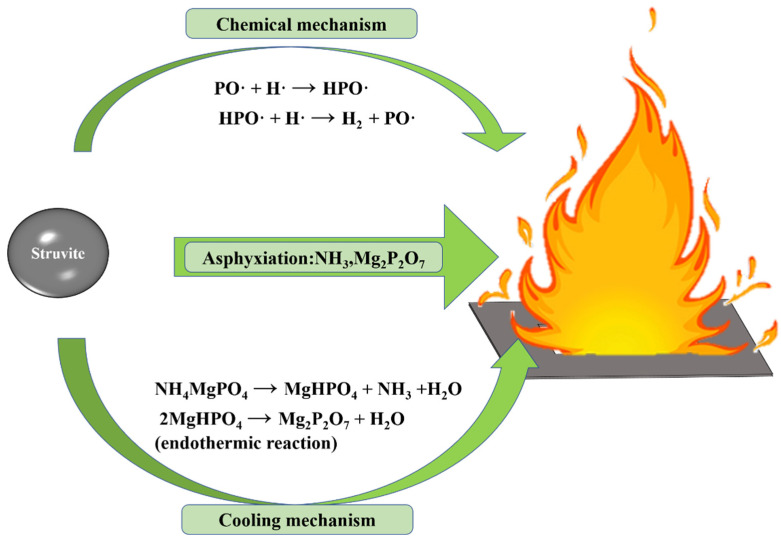
The fire-fighting mechanism of ultrafine struvite powder.

In order to explore the fire extinguishing mechanism of ultrafine struvite powder, it is explained from three aspects (Figure 12): chemical effect, asphyxiation, and cooling effect.

● Chemical mechanism

The thermal decomposition of struvite releases N-H and P-O radicals, which can absorb OH· and H· radicals in the flame [29,30]. At the same time, due to the pyrolysis of struvite, it has a larger specific surface area and greater adsorption energy, which can instantaneously absorb free radicals and effectively consume the free radicals OH· and H· in the combustion reaction. Therefore, a large number of free radicals supporting combustion in the fire field are captured, resulting in the interruption of the combustion reaction.

● Asphyxiation

Struvite (chemically bonded phosphate ceramics) releases water and ammonia at high temperatures, which dilutes oxygen and combustible gases in the fire so that the oxygen concentration is lower than the oxygen threshold of combustible gas combustion. In addition, the final product of struvite (Mg_2_P_2_O_7,_ heat-resistant ceramics) will form a dense isolation layer [15,19,31], blocking the contact between combustibles and air to achieve physical asphyxiation. Compared with NH_4_H_2_PO_4_, the final pyrolysis product of struvite is magnesium pyrophosphate (pH > 7), which is non-corrosive to metal equipment.

● Cooling mechanism

Since struvite absorbs more heat than NH_4_H_2_PO_4_ and the pyrolysis process is more concentrated and rapid, it can absorb more heat in a short time to reduce the fire temperature. Therefore, the cooling effect of struvite is particularly prominent.

## 4. Conclusions

Facing the excessive production of struvite, this paper expands the fire safety application field of struvite (chemically bonded phosphate ceramics) through economic and environmental protection methods. A simple ultrafine treatment was used to create ultrafine struvite powder. Then it was compared with NH_4_H_2_PO_4_ produced by the same process. At the same time, the fire extinguishing effect of the two samples was tested using a 1 m^3^ small fire extinguishing platform. The results are as follows:

1. The ultrafine struvite powder and ultrafine NH_4_H_2_PO_4_ powder prepared by the same process were compared by BET and SEM. It was found that the struvite sample had a smaller particle size distribution and a larger specific surface area, which was more suitable for fire extinguishing.
2. Through TG-DSC, it was found that the pyrolysis process of ultrafine struvite powder was relatively concentrated, and the decomposition efficiency was faster. More importantly, its heat absorption (458.4 J/mg) was much larger than that of NH_4_H_2_PO_4_ (156.4 J/mg), which meant that struvite could quickly reduce the fire temperature to achieve the purpose of extinguishing fire.
3. It was found that the fire extinguishing performance of struvite was better than that of NH_4_H_2_PO_4_ through small fire extinguishing experiments. Considering that struvite is a cheap sewage treatment product, it used as a cost-effective fire extinguishing agent base material agent.

## Figures and Tables

**Table 1:** Particle size and BET of two samples.

Sample	D50 (μm)	D90 (μm)	S_BET_ (m^2^/g)
**ultrafine NH_4_H_2_PO_4_**	8.961	18.625	13.64
**ultrafine struvite**	5.132	20.067	25.72

**Table 2:** Extinguishing performance and Temperature drop.

Sample	Extinction Time (s)	Mass Consumed (g)	Temperature Variation (℃)
**ultrafine NH_4_H_2_PO_4_**	13	18.625	<5
**ultrafine struvite**	10	13.067	66

**Table 3:** The characteristics test results of the above two ultrafine dry powders.

Sample	D50 (μm)	D90 (μm)	Bulk Density (g/cm^3^)	Fluidity (g/s)	Contact Angle (°)	Consumption (g)	Fire Extinguished Time (s)
**NH_4_H_2_PO_4_**	8.96	18.62	0.51	0.04	90.89	18.625	13
**Struvite**	5.13	20.06	0.51	0.04	91.44	13.067	10

## Data Availability

Not applicable.

## Supplementary Materials

The following supporting information can be downloaded at: https://www.mdpi.com/article/10.3390/ma15228021/s1, Video S1: The firefighting video of NH_4_H_2_PO_4_ under 0.2 MPa; Video S2: The firefighting video of struvite under 0.2 MPa.
